# Toxic Interplay Between Small Dipeptide Protein Repeats and Tau in Tauopathies

**DOI:** 10.1002/alz.093234

**Published:** 2025-01-03

**Authors:** Nemil Bhatt, Nicha Puangmalai, Cynthia Jerez, Nikita Shchankin, Rakez Kayed

**Affiliations:** ^1^ University of Texas Medical Branch, Galveston, TX USA

## Abstract

**Background:**

Tauopathies, including Alzheimer’s Disease and Frontotemporal Dementia, are characterized as intracellular lesions composed of aggregated tau proteins. Soluble tau oligomers are shown to be one of the most toxic species and are responsible for the spread of tau pathology. Recent studies have found that several proteins such as amyloid b, a‐synuclein, and TDP‐43 can aggregate tau. In this study, we investigated the ability of small metabolites like *C9orf72* associated dipeptide protein repeats (DPRs) to interact with and aggregate tau to form toxic soluble tau oligomers.

**Method:**

We have developed various models which express dipeptide protein repeats to understand the interaction between short peptides and tau. The dipeptide protein repeat induced tau aggregates were characterized using biophysical, as well as biochemical assays in vitro and in cellular models. Furthermore, we evaluated their toxicity, and seeding potency to understand the biological effects of this interaction.

**Result:**

Our results suggest the propensity for DPRs, especially glycine‐arginine and proline‐arginine repeats to form oligomeric structures which interact and seed tau in a prion like fashion. This leads to the production of tau oligomers causing alterations in the microtubule dynamics in cell lines as well as primary neuronal culture systems.

**Conclusion:**

Many studies have investigated the toxicity of small protein repeats, however, the role of DPR oligomers in inducing tau aggregation is still unclear. Thus, the ability to understand the toxic interplay between small peptide repeats and tau oligomers has great potential to further the understanding of tau progression and aid in the development of targeted therapeutics.

